# Time course of pupillary response to threat words before and after attention bias modification for transdiagnostic anxiety disorders: A randomized controlled trial

**DOI:** 10.1002/brb3.1664

**Published:** 2020-07-07

**Authors:** Mary L. Woody, Rachel A. Vaughn‐Coaxum, Greg J. Siegle, Rebecca B. Price

**Affiliations:** ^1^ Department of Psychiatry University of Pittsburgh Pittsburgh PA USA

**Keywords:** anxiety, attention bias modification, pupillometry, threat processing

## Abstract

**Introduction:**

Altered attention to threatening stimuli at initial and sustained stages of processing may be dissociable dimensions that influence the development and maintenance of transdiagnostic symptoms of anxiety, such as vigilance, and possibly require distinct intervention. Attention bias modification (ABM) interventions were created to implicitly train attention away from threatening stimuli and have shown efficacy in treating anxiety. ABM alters neurocognitive functioning during initial stages of threat processing, but less is known regarding effects of ABM on neural indices of threat processing at sustained (i.e., intermediate and late) stages, or if ABM‐related neural changes relate to symptom response. The current study utilized pupillary response as a temporally sensitive and cost‐effective peripheral marker of neurocognitive response to ABM.

**Materials and Methods:**

In a randomized controlled trial, 79 patients with transdiagnostic anxiety provided baseline data, 70 were randomized to receive eight sessions of twice‐weekly ABM (*n* = 49) or sham training (*n* = 21), and 65 completed their assigned treatment condition and returned for post‐training assessment.

**Results:**

Among ABM, but not sham, patients, pupillary response to threat words during initial and intermediate stages decreased from pre‐ to post‐training. Pre‐ to post‐training reductions in intermediate and late pupillary response to threat were positively correlated with reductions in patient‐reported vigilance among ABM, but not sham, patients.

**Conclusions:**

All measured stages of threat processing had relevance in understanding the neural mechanisms of ABM, with overlapping yet dissociable roles exhibited within a single neurophysiological marker across an initial–intermediate–late time continuum. Pupillometry may be well suited to measure both target engagement and treatment outcome following ABM.

## Graphical abstract

Altered attention to threatening stimuli at initial and sustained stages of processing may be dissociable dimensions that influence symptoms of anxiety and require distinct intervention; therefore, the current study utilized pupillary response as a temporally sensitive and cost‐effective peripheral marker of neurocognitive response to attention bias modification (ABM) treatment. Results demonstrate that ABM is associated with reductions in pupillary response to threat words during initial and intermediate stages of processing, which were related to top‐down cognitive control processes, and that pre‐ to post‐training reductions in intermediate and late pupillary response to threat were positively correlated with ABM‐related reductions in patient‐reported anxious arousal. Findings suggest that pupillometry may be well suited to measure both target engagement and treatment outcome following ABM.

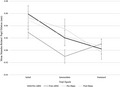

## 1 INTRODUCTION

Anxiety disorders are the most common psychiatric diagnoses (Kessler, Chiu, Demler, Merikangas, & Walters, 2005). Yet, response to frontline interventions for anxiety stands at only ~ 50%–70% (Ballenger, 2004; Barlow, Gorman, Shear, & Woods, 2000; Hofmann & Smits, 2008; McEvoy, 2007). Thus, a significant number of patients do not obtain symptom relief from well‐established treatments, resulting in a pressing need for the development and refinement of novel interventions that target mechanisms cutting across transdiagnostic anxiety disorders.

Heightened attentional bias (AB) to threat is proposed as one mechanism underlying the development and maintenance of transdiagnostic anxiety symptoms, such as vigilance (Mogg & Bradley, 2016). As a group, individuals with anxiety disorders exhibit increased attentional allocation toward threatening stimuli during initial stages of processing, which is thought to reflect greater vigilance toward threats in the environment (Bar‐Haim, Lamy, Pergamin, Bakermans‐Kranenburg, & Van Ijzendoorn, 2007). Additionally, some anxious individuals exhibit perseverative attention to threat during sustained stages of processing, which can maintain negative affect and worry (Brosschot, Gerin, & Thayer, 2006; Burkhouse, Woody, Owens, & Gibb, 2015; Price et al., 2013). Although biases at each stage of attentional processing are present simultaneously in many patients, the temporal stages of AB are posited as dissociable dimensions of threat processing that exert unique influences on the development and maintenance of anxious vigilance and possibly require distinct interventions (Price et al., 2018; Woody et al., 2019).

Attention bias modification (ABM) is a promising treatment approach that seeks to alter AB to threat through a computer‐based attention training protocol (MacLeod & Clarke, 2015). Typical ABM treatments train patients to preferentially attend to nonthreatening stimuli, rather than threatening stimuli, during *initial* stages of processing. ABM yields small‐to‐medium effects across patients with transdiagnostic anxiety disorders (Hakamata et al., 2010; Heeren, Mogoașe, Philippot, & McNally, 2015; Linetzky, Pergamin‐Hight, Pine, & Bar‐Haim, 2015). However, there is significant variability in the magnitude of patients’ symptom relief following ABM (Price et al., 2016), suggesting critical individual differences in how patients respond to ABM, which impact its effectiveness.

Theorists have suggested that ABM may have superior anxiolytic effects among patients who experience reductions in neural reactivity to threat following ABM (Wiers & Wiers, 2017). Studies examining ABM‐related neural changes among anxious patients have shown that ABM increases neurocognitive markers of attentional control [i.e., N2 event‐related potential (ERP) component; ventromedial prefrontal cortex (vmPFC) and orbitofrontal cortex (OFC) activation] and decreases neurocognitive markers of emotion processing [i.e., P2/3 ERP components; insula, subgenual anterior cingulate cortex (sgACC), and amygdala activation] (Eldar & Bar‐Haim, 2010; Taylor et al., 2013). However, past research has largely focused on group‐level effects of ABM, with less attention to individual differences in ABM‐related neural changes and relations to ABM symptom response. Further, prior research has focused on ABM‐related neural changes occurring within the first 5,000 ms of threat processing. Yet, research demonstrates that while behavioral indices of attention are typically resolved within the first 4,000 ms of processing, a sustained form of clinically relevant threat processing can occur at the neural level during intermediate (4,000–9,000 ms) and late (>9,000 ms) stages (Price et al., 2013; Siegle, Steinhauer, Carter, Ramel, & Thase, 2003; Siegle, Steinhauer, Thase, Stenger, & Carter, 2002; Siegle, Thompson, Carter, Steinhauer, & Thase, 2007). Thus, individual differences in neural changes across initial and sustained (intermediate, late) stages of processing may capture critical and clinically relevant information regarding individual symptom response to ABM.

To address limitations of prior research, the current study asked (a) whether ABM was related to neural changes in initial and sustained (intermediate, late) stages of processing of threatening stimuli, indexed by a neurophysiological marker (pupillometry), and (b) if these neural changes could be linked to ABM‐related symptom response. Pupillometry was selected to index ABM‐related neural changes for several reasons. First, pupillometry provides a cost‐effective (and thus, potentially clinically portable), temporally sensitive measure of cognitive–affective responding. Second, the pupil dilates in response to emotionally salient stimuli and under high cognitive load, thus representing an appropriate measure of ABM target engagement. Finally, the pupil is innervated by the same limbic and prefrontal brain regions implicated in ABM‐related neural changes (Graur & Siegle, 2013).

The current study was a secondary analysis of a previously described study (Price et al., 2018; Price, Woody, Panny, & Siegle, 2019; Woody et al., 2019). Of note, the current study was not designed and optimized to test group‐level effects of ABM (relative to sham). Instead, the study was intended to maximize sensitivity for identifying individual differences in ABM efficacy. Thus, our analytic plan did not include testing group‐level effects and instead focused on the active ABM group, using effect sizes and significance levels from the control group as an exploratory probe of the specificity of findings to active ABM. Patients were recruited across multiple diagnostic categories of anxiety, as transdiagnostic samples are thought to best represent real‐world patient populations and present opportunities to test cross‐cutting mechanisms. Using a randomized controlled design, patients were assigned to either ABM or sham training. Patients completed an emotion processing task pre‐ and post‐training, quantifying pupillary responses to threat and neutral words during initial, intermediate, and late stages of processing. Because reductions in pupillary response to emotional stimuli are thought to reflect reductions in a summative index of cognitive and affective processing load (Graur & Siegle, 2013), we hypothesized that ABM (but not sham) would be related to reductions in pupillary response to threat words at initial stages of processing (Wiers & Wiers, 2017), while also investigating the possibility that sustained stages of processing might be impacted. Finally, because greater pupillary response to threatening stimuli is related to more anxiety symptoms and disorders at both initial and sustained stages of processing (Cascardi, Armstrong, Chung, & Paré, 2015; Hepsomali, Hadwin, Liversedge, & Garner, 2017; Kret, Stekelenburg, Roelofs, & De Gelder, 2013; Price et al., 2013), we predicted that all stages of threat processing might represent neural mechanisms by which ABM exerts clinical impact. We therefore expected ABM (but not sham) patients who exhibited the greatest reductions in pupillary response to threat stimuli at any stage of processing to be among the patients who exhibited the most symptom relief from anxious vigilance.

## 2 MATERIALS AND METHODS

### 2.1 Participants

**Figure 1:**
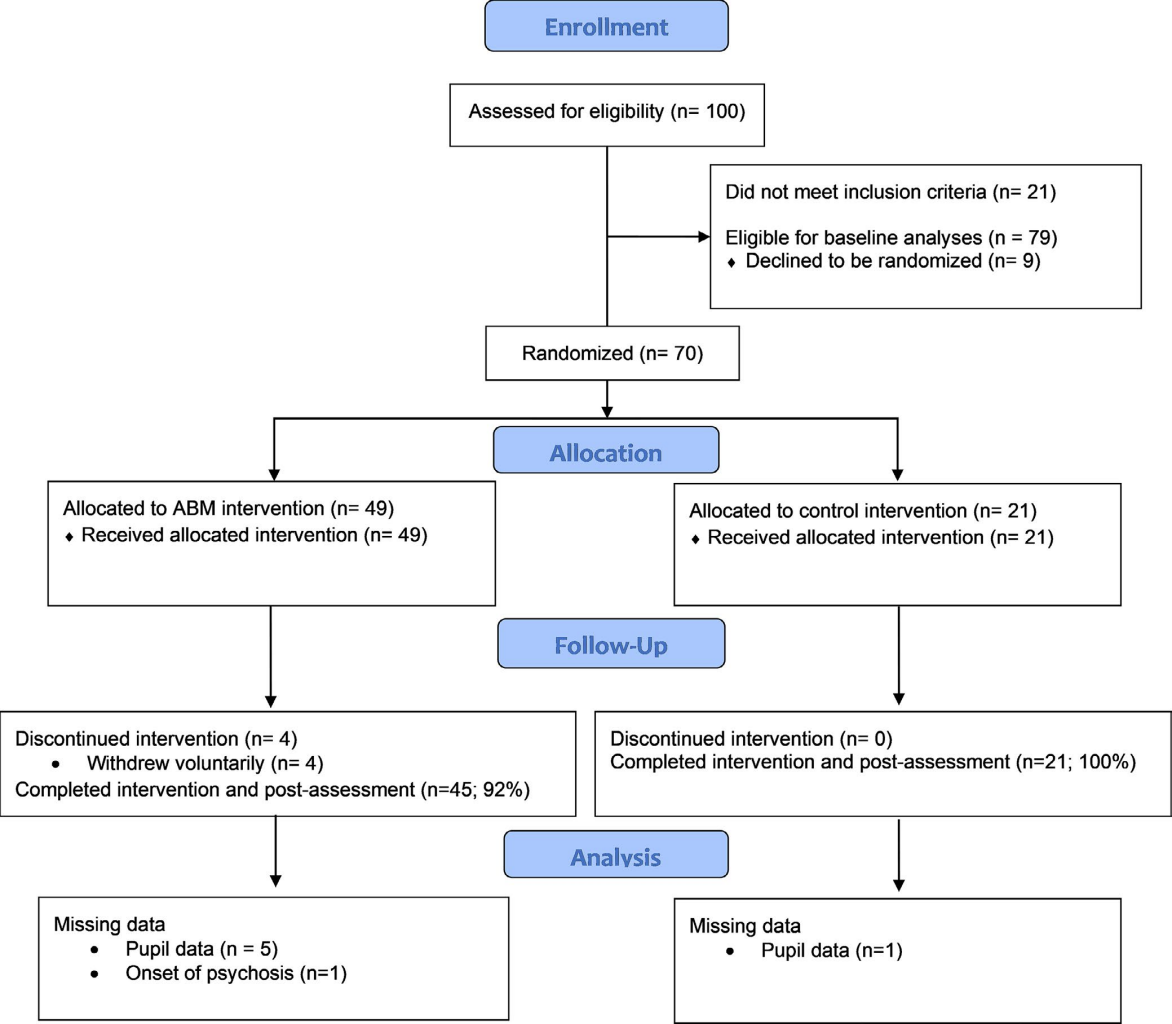
CONSORT diagram

Seventy‐nine unmedicated adults reporting clinical levels of transdiagnostic anxiety and associated clinician‐rated disability provided baseline data, and 65 of those patients completed ABM (*n* = 44) or sham (*n* = 21) training. See Figure 1 (CONSORT diagram; clinicaltrials.gov: NCT02303691) for details regarding enrollment, allocation, follow‐up, and missing data and Table 1 for demographic and clinical characteristics. All patients provided informed consent prior to their inclusion in the study, and all study procedures were approved by the University of Pittsburgh's Institutional Review Board.

**Table 1:** Demographic and clinical characteristics of the sample

	ABM			Sham		
	Pretraining	Post‐Training	Pre–post effect size [95% CI]	Pretraining	Post‐Training	Pre–post effect size [95% CI]
Demographics						
Caucasian, *n* (%)	28 (64%)	‐	‐	15 (75%)	‐	‐
Female, *n* (%)	34 (77%)	‐	‐	16 (80%)	‐	‐
Age	30.61 (9.28)	‐	‐	29.04 (11.02)	‐	‐
Primary outcome measures						
MASQ: Anxious Arousal	32.43 (10.79)	28.16 (9.40)	***d* = −0.42*^d^*** **[−0.63 to −0.22]**	34.05 (10.72)	30.29 (13.92)	*d* = −0.30*^c^* [−0.67 to 0.07]
CAPS: Vigilance	4.61 (2.00)	4.02 (1.97)	***d* = −0.30*^c^*** **[−0.55 to −0.05]**	5.24 (2.23)	4.19 (2.16)	***d* = −0.48*^d^* [−0.84 to −0.11]**
Pupil to Threat E1	0.06 (0.09)	0.03 (0.06)	***d* = −0.38*^d^*** **[−0.67 to −0.09]**	0.05 (0.08)	0.05 (0.06)	*d* = −0.06*^a^* [−0.58 to 0.47]
Pupil to Threat E2	0.02 (0.08)	−0.01 (0.07)	***d* = −0.41*^d^*** **[−0.76 to −0.05]**	0.03 (0.06)	0.02 (0.07)	*d* = −0.18*^b^* [−0.77 to 0.42]
Pupil to Threat E3	0.00 (0.05)	0.01 (0.05)	*d* = 0.17*^b^* [−0.27 to 0.61]	0.00 (0.05)	0.01 (0.06)	*d* = −0.18*^b^* [−0.05 to 0.03]
Pupil to Neutral E1	0.04 (0.06)	0.02 (0.05)	***d* = −0.41*^d^*** **[−0.74 to −0.08]**	0.06 (0.06)	0.05 (0.07)	*d* = −0.03 *^a^* [−0.48 to 0.42]
Pupil to Neutral E2	0.00 (0.06)	−0.01 (0.06)	*d* = −0.20*^b^* [−0.58 to 0.19]	0.02 (0.08)	0.03 (0.07)	*d* = −0.07*^a^* [−0.39 to 0.53]
Pupil to Neutral E3	−0.01 (0.06)	−0.02 (0.06)	*d* = −0.17*^b^* [−0.54 to 0.20]	0.00 (0.07)	0.01 (0.06)	*d* = 0.15*^b^* [−0.40 to 0.69]

Note.

Data presented as mean (*SD*) unless otherwise noted.

Abbreviations: CAPS, Clinician‐Administered PTSD Scale; CI, Confidence Interval; E, epoch; ES, Effect Size; MASQ, Mood and Anxiety Symptoms Questionnaire.

Each ES is interpreted based on Cohen's “Rules‐of‐Thumb” and is assigned a symbol to indicate magnitude (*^a^* = negligible; *^b^* = small; *^c^* = small‐medium; *^d^* = medium). If the 95% CI for the ES includes “0”, it indicates statistical nonsignificance. “Significant” ES is bolded.

### 2.2 Measures

#### 2.2.1 ABM and sham conditions

As described in detail in previous publications from this sample (Price et al., 2018, 2019; Woody et al., 2019), patients and clinical assessors were blind to treatment assignment, and the ABM and sham conditions were modeled after prior studies (Amir, Beard, Burns, & Bomyea, 2009). Briefly, patients in both conditions completed a modified dot‐probe task to retrain attention to threat and neutral words through eight, twice‐weekly laboratory‐based sessions. During training trials, word pairs (80% threat–neutral; 20% neutral–neutral) were presented vertically for 500 ms, followed by a probe in either the upper‐ or lower‐word location. Patients responded to indicate the probe location. In ABM, 100% of threat–neutral trials had the probe replace the neutral word in a threat–neutral pair, shaping attention away from threatening cues through practice. In the sham condition, the probe replaced either the threat or neutral word with equal likelihood.

#### 2.2.2 Clinical outcome measures

Given ABM’s target mechanism of threat vigilance, primary symptom‐level outcome measures were designed to assess self‐reported and clinician‐rated involuntary orientation toward potential threats and concomitant anxious arousal symptoms (i.e., vigilance/hypervigilance‐related symptoms). The primary self‐report outcome was the Anxious Arousal subscale from the Mood and Anxiety Symptoms Questionnaire (MASQ; 64‐item short form) (Clark & Watson, 1991; Watson et al., 1995), which captures clinically relevant symptoms of anxious vigilance within transdiagnostic disorders. The primary clinician‐rated outcome for the trial was the “hypervigilance” item of the well‐validated Clinician‐Administered PTSD Scale [CAPSvigilance; (Blake et al., 1995)], which sums two subitems assessing frequency and intensity of vigilance (e.g., “have you been especially alert or watchful” for threat‐related information or “felt as if you were constantly on guard?”). For additional details and psychometric properties of these measures, as well as information regarding secondary outcome inclusion and analysis, see Supplement and prior work from our group (Price et al., 2018).

Outcome measures were collected at two timepoints: a pretraining baseline visit (approximately 1–2 weeks prior to the beginning of attention training) and a post‐training visit (within approximately 1 week of completing the final training session). Residual symptom scores post‐training (regressing out pretraining scores) were calculated within each treatment group; lower numbers indicate fewer residual symptoms (more favorable outcome) relative to other individuals in the same treatment group. Residual scores were chosen in lieu of change scores because they generally improve power and account for nonstatic relations between pre and post scores (Petscher & Schatschneider, 2011).

#### 2.2.3 Self‐Referent Encoding Task (SRET)

The SRET was adapted from Siegle and colleagues (Siegle, Steinhauer, Carter, et al., 2003; Siegle et al., 2002) and was administered both at the baseline and post‐training visit. The SRET is designed to capture protracted cognitive–affective processing that persists in the aftermath of stimulus presentation. Specifically, participants were instructed to respond to one of two prompts: “Does it worry you?” or “Is it relevant for you?” by pressing a button to indicate one of three options: Yes, Somewhat, or No. Each prompt was used for the entirety of one of two task blocks (one block = 30 trials; block order counterbalanced across participants), and reminders of the prompt and the button‐press options were displayed in the upper right corner throughout the entire block. Each trial commenced with a forward mask (row of “X’s”) displayed for 1,000 ms, followed by presentation of a threat or neutral word for 300 ms, followed by a backward mask (row of “X’s”) for the remainder of the 12,000‐ms period. Both threat (*n* = 30) and neutral (*n* = 30) word trials were presented in random order. Task stimuli were drawn from the patient's word lists, which consisted of 40 idiographic words chosen collaboratively by the patient and the clinical assessor and 20 normative words used across all patients.

During the SRET, pupil size was recorded using a table‐mounted RK‐768 eye tracker, consisting of a video camera and infrared light source pointed at a participant's eye and a device that tracked the location and size of the pupil and corneal reflection at 60 Hz (every 16.7 ms). Data were cleaned using standard procedures (Siegle, Ichikawa, & Steinhauer, 2008). Linear interpolation was used to replace blinks throughout the data set, and the data were smoothed using a 10‐point weighted average filter. The average pupil diameter over the 333 ms preceding the onset of the trial was subtracted from pupil diameter after trial onset to produce stimulus‐related pupil dilation. Based on previous research (Price et al., 2013; Siegle, Granholm, Ingram, Matt, 2001; Siegle et al., 2002; Siegle et al., 2007), mean stimulus‐related pupil dilation for each of three a priori time epochs of interest was calculated by averaging pupillary response across initial (1,000–4,000 ms), intermediate (4,001–9,000 ms), and late (9,001–12,000 ms) trial epochs for each emotion condition (threat, neutral). Of note, although the initial epoch contains both peri‐stimulus and early poststimulus processing, the interpretation of pupillary response at initial, intermediate, and late epochs is fairly consistent as an index of protracted processing. Pupillary response did not differ across “Did it worry you?” versus “Is it relevant for you?” prompts or idiographic versus normative words (see Supplement and Table S1), and thus, pupillary response was collapsed across prompt and word type conditions.

An additional *post hoc* analysis of pupil data was used to aid in interpreting pupil findings through neural “source localization” in a subset of patients (*n* = 62) with usable functional magnetic resonance imaging (fMRI) data collected during an identical SRET administered at baseline (see Supplement and Figure S1).

### 2.3 Analytic plan

#### 2.3.1 Baseline analyses

To probe patients’ pretraining neurocognitive profile, analyses were performed to determine differences in the time course of pupillary response to threat versus neutral words among the 79 patients with *baseline* data.

#### 2.3.2 Pre‐ to post‐training completer analysis

Completer analysis examining *pre‐ to post‐training* effects was first performed among ABM patients (*n* = 44), with significant analyses repeated among sham patients (*n* = 21). This statistical approach was selected for several reasons. The project's conceptual focus was not on examining group differences between ABM and sham participants, and instead, the preregistered specific aims of the grant were to examine (a) neural mechanisms correlated with behavioral manifestations of initial and sustained attention to threat at baseline; (b) ABM effects on symptom‐level, behavioral, and neural dimensions of initial and sustained threat processing; and (c) associations between baseline neural dimensions and ABM outcomes (K23MH100259; clinicaltrials.gov: NCT02303691). To maximize power for these specific aims focused on mechanisms underlying ABM response, the study design included uneven allocation to the ABM versus sham condition; thus, preserving an a priori focus on the ABM group enhances statistical power to characterize the ABM sample consistent with primary study aims. The sham condition was included to probe specificity of results to ABM through effect size comparison, consistent with prior publications from this sample (Price et al., 2018, 2019; Woody et al., 2019).

## 3 RESULTS

### 3.1 Baseline analyses

**Figure 2:**
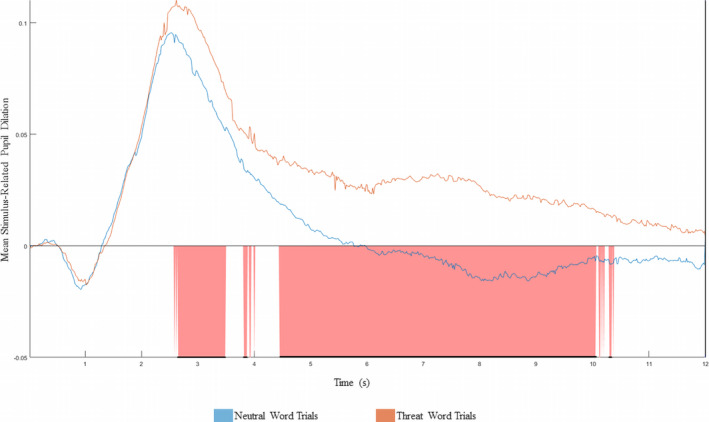
Differences between pupillary response to threat versus neutral words across the time course. Mean stimulus‐related pupil dilation is plotted across the 12,000‐ms trial for both neutral and threat words. Significant pairwise differences are highlighted in red below the axis with bolded black lines showing time regions with enough consecutive tests (>3) to be considered significant (*p* < .05). See also Supplement and Figure S2

To determine the effect of Emotion (threat, neutral) and Epoch (initial, intermediate, late) conditions on pupillary response during the SRET at baseline, we conducted a 2(Emotion) × 3(Epoch) repeated measures analysis of variance (ANOVA) with mean stimulus‐related pupil dilation serving as the dependent variable. In addition to main effects of Emotion, *F*(1,78) = 5.55, *p *= .02, $\eta_{p}^{2}$ **= 0.07, and Epoch, *F*(2,77) = 30.74, *p*<.001, $\eta_{p}^{2}$ **= 0.28, there was an Emotion × Epoch interaction, *F*(2,77) = 3.67, *p *= .03, $\eta_{p}^{2}$ **= 0.05. To probe this interaction, we compared pupillary response for threat versus neutral words separately for each of the Epoch conditions. Follow‐up analyses revealed that pupillary response was higher to threat, versus neutral, words during initial, *F*(1,78)* *= 4.38, *p *= .04, $\eta_{p}^{2}$ **= 0.05, and intermediate, *F*(1,78)* *= 9.77, *p *= .002, $\eta_{p}^{2}$ **= 0.11, but not late epochs, *F*(1,78)* *= 1.53, *p *= .22, $\eta_{p}^{2}$ **= 0.02. We also conducted comparative supplementary analyses examining differences in baseline pupillary response to neutral and threat word trials across a continuous time course (see Figure 2, Supplement, and Figure S2.). Findings were largely consistent, providing additional support for the a priori defined initial–intermediate–late continuum.

### 3.2 Pre‐ to post‐training completer analysis

**Figure 3:**
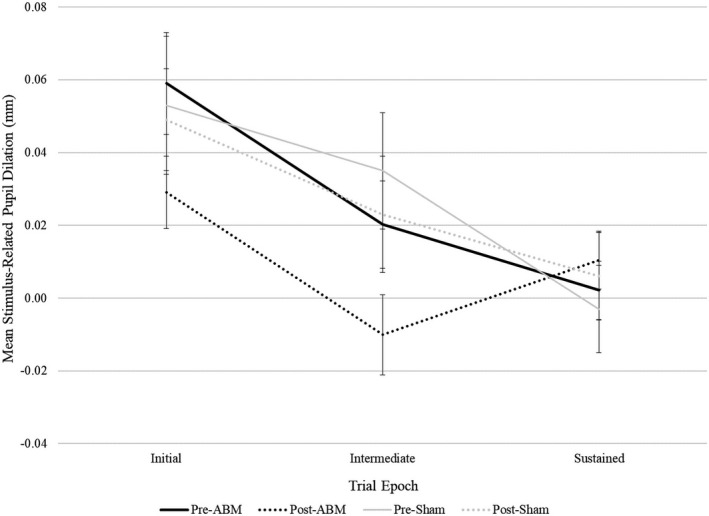
Change in pupillary response to threat words from pre‐ to post‐training

To examine pre‐ to post‐ABM change in pupillary response across ABM patients, we conducted a 2(Emotion) × 3(Epoch) × 2(Visit) repeated measures ANOVA with mean stimulus‐related pupil dilation serving as the dependent variable. Results of these analyses are presented in Table 2. To probe the form of the highest‐order Visit × Emotion×Epoch interaction, we examined the Visit × Epoch interaction separately for each of the Emotion conditions. For pupillary response to threat words, there was a Visit × Epoch interaction, *F*(2, 37) = 8.22, *p *= .001, $\eta_{p}^{2}$ **= 0.18. Follow‐up analyses revealed that pupillary response to threat decreased from pre‐ to post‐training during initial, *F*(1,38) = 7.31, *p *= .01, $\eta_{p}^{2}$ **= 0.16, and intermediate, *F*(1,38) = 5.51, *p *= .02, $\eta_{p}^{2}$ **= 0.13, but not late epochs, *F*(1,38) = 0.56, *p *= .46, $\eta_{p}^{2}$ **= 0.02. For pupillary response to neutral words, the Visit × Epoch interaction was not significant, *F*(2,37) = 0.64, *p *= .53, $\eta_{p}^{2}$ **= 0.02. Finally, to explore specificity of these findings to ABM, we repeated analyses among sham patients. There were no significant pre‐ to postchanges in pupillary response among sham patients, even when taking an exploratory approach of testing each epoch at each valence in an independent pre–post *t*‐test. Effect size specificity comparisons for significant analyses revealed that for ABM patients, effect sizes for pupillary changes to threat words during initial and intermediate epochs were of medium magnitude (*d*s ranged from −0.38 to −0.41), whereas, for sham patients, effect sizes were negligible to small (*d*s ranged from −0.06 to −0.18) (see Table 1 for all effect sizes and CIs in both training conditions and Figure 3 for visual depiction; see also Table S2 for an omnibus 2(Group) × 2(Visit) × 2(Emotion) × 3(Epoch) repeated measures analysis of variance (ANOVA) with mean stimulus‐related pupil dilation serving as the dependent variable).

**Table 2:** Results of the repeated measures ANOVAs predicting prospective changes in pre‐ to post‐training mean stimulus‐related pupil dilation as a function of emotion and epoch conditions

	*F*
Emotion	2.72
Epoch	16.44*
Visit	8.54*
Emotion × Epoch	0.33
Emotion × Visit	0.02
Epoch × Visit	3.82*
Emotion × Epoch ×Visit	3.81*

* *p* < .05. **p < .01. ***p < .001.

Hierarchical linear modeling (HLM) was used to test our hypothesis that patients who exhibited greater symptom improvement in response to ABM would also exhibit the greatest reductions in pupillary response to threat words from pre‐ to post‐training**.** Within each model, mean stimulus‐related pupil dilation at post‐training was included as the outcome variable, and primary outcome residual scores and pretraining pupillary response were added individually as between subject factors. Separate models were run for pupillary response for each emotion and epoch condition for several reasons. First, regression‐based analyses that examine valence or epoch effects separately can provide useful information regarding cognitive–affective processing biases relevant to ABM efficacy (Beevers, Clasen, Enock, & Schnyer, 2015; Kret et al., 2013; Price et al., 2018; Woody et al., 2019). Second, this approach reduces the statistical assumptions and complexity required of HLM models and also allows for easier comparisons with a previous publication from the project, which utilized HLM (Price et al., 2019).

Among ABM patients, lower MASQ anxious arousal residual scores were related to reduced pupillary response to threat words during intermediate, *t*(36) = 2.40, *p *= .02, *r*_partial_ = 0.38, and late epochs of the trial, *t*(36) = 2.22, *p *= .03, *r*_partial_ = 0.36, suggesting that individuals with greater reductions in self‐reported anxious arousal exhibited reduced pupillary response to threat words during sustained stages of processing. In contrast, this effect was not significant in the initial epoch for threat words (*p *= .29). To explore specificity of these findings to ABM, we repeated significant analyses among sham patients. Changes in pupillary response to threat words during intermediate and late epochs were not significantly related to self‐reported symptom response in sham patients (lowest *p *= .37), and effect sizes were uniformly small (*r*_partial_ ≤ 0.20).

For neutral words, lower MASQ anxious arousal residual scores were related to reduced pupillary response during initial epochs of the trial, *t*(36) = 2.46, *p *= .02, *r*_partial_ = 0.39, but not during intermediate or late epochs (lowest *p *= .50). Specificity analyses showed that change in pupillary response to neutral words during the initial epoch was not significantly related to self‐reported symptom response in sham patients (*p *= .25), but the effect was small‐to‐medium (*r*_partial_ = 0.26).

These effects did not generalize to a clinician‐rated measure. Among ABM patients, CAPS‐vigilance residual scores were not significantly related to pupillary response across any of the emotion or epoch conditions (lowest *p *= .06).

## 4 DISCUSSION

The primary aim of the current study was to assess the temporal course of ABM‐related changes in a neurocognitive marker of threat processing (pupillometry) and determine whether individual differences in reductions in threat processing would be associated with ABM‐related symptom change in a transdiagnostic group of anxious patients. Findings showed that ABM, relative to sham, was associated with reductions in pupillary response to threat stimuli during initial and intermediate stages of processing. Notably, these reductions were well matched to the group's temporal pattern of elevated pupillary responses to threat at baseline, suggesting that ABM is mechanistically suited to modify the baseline neurocognitive profile displayed by transdiagnostic anxious patients as a group. However, when examining individual differences in treatment response, the temporal characteristics of findings shifted slightly. Specifically, findings revealed that individuals who exhibited reductions in pupillary response to threat words during sustained (intermediate, late), but not initial, stages of processing were more likely to report better symptom response. Together, these findings suggest that all measured stages of threat processing had relevance in understanding the neural mechanisms of ABM, with overlapping yet dissociable roles exhibited within a single neurophysiological marker across an initial–intermediate–late time continuum.

Findings contribute to a growing body of research suggesting that, at the group‐level, ABM alters neurocognitive markers of emotion processing and attentional control during threat processing (Wiers & Wiers, 2017). In the current study, we assessed threat processing via pupillometry given that the pupil receives afferent inputs from both prefrontal and limbic regions (Graur & Siegle, 2013), thereby providing a summative index of activation in brain regions associated with ABM‐related neural changes. In a supplementary analysis conducted among a subset of patients who performed an identical SRET at baseline during functional neuroimaging, pupil values during initial stages of threat processing tracked with larger responses in a right middle frontal gyrus region implicated in cognitive control and modulating attention for emotional stimuli (Banich et al., 2009; Nee, Wager, & Jonides, 2007; Siegle, Steinhauer, Stenger, Konecky, & Carter, 2003). This suggests that the ABM‐associated reductions in pupillary response to threat in the current study may better reflect top‐down cognitive control processes and/or cognitive load rather than salience circuit driven arousal or affective responding, which is consistent with prior work showing that ABM increases brain activation associated with attentional control during initial phases of processing (Eldar & Bar‐Haim, 2010; Taylor et al., 2013), including middle frontal gyrus (Beevers et al., 2015). Because prior work did not thoroughly examine sustained stages of processing, the current study provides preliminary evidence that ABM is associated with group‐level reductions during intermediate stages as well but did not extend robustly to late stages.

Study results suggest that ABM‐related reductions in pupillary response during initial stages may not be related to symptom change across individuals. It is possible that change during the initial stage was impacted by ceiling effects given that ABM exerted a group‐level effect on the initial epoch and is designed specifically to reallocate attentional resources away from threat during initial stages of processing. Because ABM does not provide strategies for processing threat stimuli during sustained stages, there likely was more individual variability in cognitive–affective load at intermediate and late stages. For some ABM patients, the skills learned during initial stages of threat processing may have generalized, leading to reduced cognitive–affective load across the time course, whereas others were not able to translate these skills to sustained stages. Because protracted processing of threat is thought to maintain worry and negative affect (Brosschot et al., 2006; Burkhouse et al., 2015), patients who exhibited more generalized reductions in biases following ABM may have experienced greater relief from symptoms.

Results from the current study are complemented by other published findings from this project (K23MH100259; clinicaltrials.gov: NCT02303691) and, together, describe how ABM efficacy may occur at an individual level among patients with transdiagnostic anxiety. Our research utilizing fMRI has suggested that there are at least two mechanistic neural predictors of ABM efficacy. First, because the goal of ABM is to target initial attentional vigilance to threat, patients who display larger transient attentional responses to negative cues across a range of cognitive–affective brain regions (e.g., ventrolateral prefrontal cortex, anterior cingulate cortex, amygdala) at baseline exhibit superior improvements in anxiety following ABM (Price et al., 2018). Second, because active ABM interventions systematically redirect attention toward neutral stimuli, individuals who demonstrate protracted levels of amygdala activity in the aftermath of neutral words at baseline are more likely to be poor mechanistic candidates for ABM (Woody et al., 2019). Other reported analyses focused on dissecting subcomponents of attention at the behavioral level, revealing behavioral attentional patterns that may confer a good mechanistic match to ABM (Price et al., 2019). The current study utilized pupillometry as a peripheral marker of neural function, collected both before and after ABM or sham training, and thus substantively extends prior findings. Specifically, while previous published papers from this project utilized fMRI indices, collected from the full sample only at baseline, to assess neural *predictors* of treatment response, the current findings determine how neural indices may *change* across the course of treatment. Because pupillometry is a cost‐effective, time‐sensitive index of cognitive–affective processing, it provides complementary information to fMRI, which is often too cost‐prohibitive to be collected at repeated timepoints and has limited applicability in clinical settings. Results from the current study demonstrate that the same neural index that predicted ABM efficacy (initial neural reactivity to threat) was also reduced effectively and robustly by ABM. However, the heterogenous impact of ABM on sustained attentional components was revealed to be associated with individual differences in treatment outcomes. Critically, these findings highlight the unique benefit of pupillometry due to its temporal specificity and cost‐effectiveness, suggesting it is well suited to index both target engagement and treatment outcome following ABM.

Although the current study benefited from random assignment to ABM or sham, the conceptual focus on examining individual differences in ABM efficacy resulted in an uneven allocation protocol that favored ABM and reduced power to examine pupillary changes in the sham condition. While our effect size comparisons provide preliminary evidence that changes in pupillary response were specific to ABM relative to sham, future research would benefit from larger samples to further test placebo effects and examine the specific effects of intervention on pupillary response for ABM compared to frontline interventions (e.g., CBT). The impact of the current study is also limited by its examination of ABM‐related neural changes in a controlled laboratory setting; future work is needed to determine how pupillary response could inform intervention research in a clinical setting. This avenue of future research could be transformative in integrating cognitive neuroscience into psychiatry clinics of the future, given pupillometry's promise to be clinically portable in a way that other neural measures, such as fMRI, are not.

## 5 CONCLUSION

The current study supports the hypothesis that biases at each stage of threat processing are relevant toward understanding the neural mechanisms of ABM and exert overlapping yet dissociable impact on treatment response. These findings add to a burgeoning body of research that suggests more robust generalization across the full temporal gradient of threat processing may be key to maximizing clinical impact (Price et al., 2018; Woody et al., 2019). Further, our findings support the utility of using pupillometry to assess ABM‐related neurocognitive outcomes. Unlike measures such as fMRI, pupillometry is noninvasive, cost‐effective, and can be administered outside of the laboratory via mobile technology by practitioners who do not need advanced expertise (Graur & Siegle, 2013). As seen in the current study, pupillometry provides an opportunity to examine change in a putative mechanism of ABM response and its relation to treatment response, with potential for use in clinical settings as a tool to track treatment response. On the horizon, research seeks to exploit pupillometry as an avenue for biofeedback on cognitive–affective processing (Ehlers, Strauch, Georgi, & Huckauf, 2016), which could provide future opportunities to tailor ABM to the neurocognitive profile of each patient by providing targeted feedback on their ABM‐related performance. Taken together, past research and the current findings emphasize the importance of examining the temporal course of ABM‐related changes in threat processing, suggesting future strategies for optimizing ABM treatment outcomes and care for patients with anxiety disorders.

## 6 DATA SHARING STATEMENT

The data that support the findings of this study are available from the corresponding author upon reasonable request.

## CONFLICT OF INTEREST

All authors report no biomedical financial interests or potential conflicts of interest.

## AUTHOR CONTRIBUTION

M.L.W., G.J.S., and R.B.P. developed the study concept and contributed to the study design. Testing and data collection were performed by R.B.P. and her staff. M.L.W., R.A.V.C., and R.B.P. performed the data analysis and interpretation. M.L.W. drafted the paper. All authors provided revisions and approved the final version of the paper for submission.

## Supporting information


**Appendix S1.**
