# Supplementary material for: Time course of pupillary response to threat words before and after attention bias modification for transdiagnostic anxiety disorders: A randomized controlled trial
Source: Brain Behav. 2020 Jul 7;10(8):e01664. doi: 10.1002/brb3.1664 (PMC7428474; doi:10.1002/brb3.1664)
Supplement: Supplementary file 1 — Appendix S1. [file BRB3-10-e01664-s001.docx]

**Time Course of Pupillary Response to Threat Words Before and After Attention Bias Modification for Transdiagnostic Anxiety Disorders: A Randomized Controlled Trial**

***Supplemental Information***

[Supplemental Materials and Methods 2](#_Toc35527530)

[Inclusion/Exclusion Criteria 2](#_Toc35527531)

[Psychometric Properties of Primary Outcome Measures 2](#_Toc35527532)

[Secondary Clinical Outcome 3](#_Toc35527533)

[Supplemental Analyses 4](#_Toc35527534)

[The Effect of Prompt and Word Type on Pupillary Response during the SRET 4](#_Toc35527535)

[Omnibus Test of Pre- to Post-Changes in Pupillary Response during the SRET 6](#_Toc35527536)

[Neural Source Localization of Pupil Findings: fMRI Analysis 7](#_Toc35527537)

[Examination of Differences between Pupillary Response to Threat versus Neutral Words across the Time Course at Baseline 9](#_Toc35527538)

[Analysis of Secondary Clinical Outcome 11](#_Toc35527539)

[Supplemental References 12](#_Toc35527540)

# **Supplemental Materials and Methods**

## Inclusion/Exclusion Criteria

All patients displayed clinical levels of transdiagnostic anxiety at the baseline assessment as defined by two inclusion criteria. Specifically, patients had to endorse often feeling anxious, shy or worried, as defined by scores > 45 on the Spielberger State-Trait Anxiety Inventory trait form (STAI-T) (Spielberger, 2010) and exhibit clinically significant impairment as indicated by scores at or above 75th percentile on the WHO Disability Assessment Schedule 2.0 (WHODAS 2.0) (Üstün, Kostanjsek, Chatterji, & Rehm, 2010). These two criteria were designed to get an appropriate distribution of individuals with clinically impairing transdiagnostic anxiety. Patients were included in the study if they (a) passed the first two criteria; (b) were not currently participating in cognitive behavioral therapy (CBT); (c) were not taking any psychotropic medications; (d) met standard fMRI inclusion criteria; (e) had no evidence of bipolar, psychotic, autism spectrum, substance dependence, or primary depressive disorder; (f) showed no evidence of acute suicidality; (g) scored >20/40 on the Snellen test indicating normal or corrected-to-normal vision; and (h) had a reading level >6th grade as per the Wide Range Achievement Test – Revised (WRAT-R) reading scale (Jastak & Wilkinson, 1984).

## Psychometric Properties of Primary Outcome Measures

The primary self-report outcome for the trial was the Anxious Arousal subscale from the Mood and Anxiety Symptoms Questionnaire (MASQ; 64-item short form). The MASQ is a well-validated questionnaire that assesses the severity of anxious symptoms and allows discrimination between anxiety symptoms and general distress, with the latter being common across a range of internalizing and externalizing disorders (Clark & Watson, 1991; Watson et al., 1995). The Anxious Arousal subscale was of principle interest given its capacity to capture clinically relevant symptom patterns of anxious vigilance within transdiagnostic disorders. In the current study, internal consistency for the MASQ anxious arousal subscale was good (α = .88).

The primary clinician-rated outcome for the trial was the “hypervigilance” item of the well-validated Clinician-Administered PTSD Scale [CAPS-vigilance; (Blake et al., 1995)], which sums two sub-items assessing frequency and intensity of vigilance (e.g., “have you been especially alert or watchful” for threat-related information or “felt as if you were constantly on guard?”). This scale allows the clinician to tailor questions on an idiographic basis so that it can capture the construct of vigilance to threat across a wide spectrum of fear- and distress-related disorders. Scores encapsulate both frequency and distress scales, with the latter distress component being explicitly quantified irrespective of frequency so that it would flexibly capture decreased severity of vigilance even for cases where exposure to triggering events is more circumscribed. To ensure transdiagnostic relevance of this measure, assessors were trained to provide idiographic examples of vigilance that were relevant to the participant’s principle anxiety domains (e.g., were you “on guard” for signs of negative social evaluation, health/monetary/safety concerns, interoceptive panic cues, etc.). To assess inter-rater reliability, a random subset of videotaped interviews (15%) was scored by a second rater, and 100% reliability was obtained.

**Secondary Clinical Outcome**

The MASQ General Distress: Anxiety subscale was used as a secondary outcome measure to assess for levels of general distress that are specific to anxiety disorders. In the current study, the MASQ General Distress Anxiety Subscale exhibited good internal consistency (α = .83).

# **Supplemental Analyses**

## The Effect of Prompt and Word Type on Pupillary Response during the SRET

During the SRET, participants were instructed to respond to one of two prompts: “Does it worry you?” or “Is it relevant for you?” followed by the presentation of a threat or neutral words. Task stimuli were drawn from the patient’s word lists, which consisted of 40 idiographic words chosen collaboratively by the patient and the clinical assessor and 20 normative words used across all patients. To determine if Prompt (i.e., “Does it worry you?” versus “Is it relevant for you?”) or Word Type (i.e., idiographic versus normative) had an effect on pupillary response during the SRET at baseline, we conducted a 2 (Prompt Type) × 2 (Word Type) × 2 (Emotion) × 3 (Epoch) repeated measures analysis of variance (ANOVA) with mean stimulus-related pupil dilation serving as the dependent variable. The results of these analyses are presented in **Table S1**. The only significant finding regarding Prompt and Word Type was a Word Type × Prompt Type × Epoch interaction. To probe this interaction, we examined Word Type × Prompt Type interactions for each epoch separately. However, the Word Type × Prompt Type interaction was not significant at any epoch (lowest *p* = .16). Thus, pupillary response was collapsed across prompt and word type conditions for the primary study analyses.

*Table S1.* Results of the Repeated Measures ANOVA analyses examining mean stimulus-related pupil dilation during the baseline SRET as a function of Prompt Type, Word Type, Emotion, and Epoch conditions.

|  |  |
| --- | --- |
|  | *F* |
| Prompt Type | 0.64 |
| Word Type | 1.17 |
| Emotion | 5.41* |
| Epoch | 26.27*** |
| Prompt Type × Word Type | 0.00 |
| Prompt Type × Emotion | 1.13 |
| Prompt Type × Epoch | 2.01 |
| Word Type × Emotion | 1.17 |
| Word Type × Epoch | 0.36 |
| Emotion × Epoch | 2.30 |
| Prompt Type × Word Type × Emotion | 0.07 |
| Prompt Type × Word Type × Epoch | 6.87** |
| Prompt Type × Emotion × Epoch | 1.66 |
| Word Type × Emotion × Epoch | 1.67 |
| Prompt Type × Word Type × Emotion × Epoch | 0.75 |
|  |  |

**p* < .05. ***p* < .01. ****p* < .001.

## Omnibus Test of Pre- to Post-Changes in Pupillary Response during the SRET

*Table S2.* Results of the Omnibus Repeated Measures ANOVA analyses predicting changes in pre- to post-training mean stimulus-related pupil dilation as a function of ABM group, emotion, and epoch conditions.

|  |  |
| --- | --- |
|  | *F* |
| Group | 3.28 |
| Emotion | 0.94 |
| Epoch | 23.21*** |
| Visit | 1.95 |
| Group × Emotion | 1.02 |
| Group × Epoch | 1.25 |
| Group × Visit | 2.43 |
| Emotion × Epoch | 0.26 |
| Emotion × Visit | 0.12 |
| Epoch × Visit | 2.97 |
| Group × Emotion × Epoch | 0.51 |
| Group × Emotion × Visit | 0.03 |
| Group × Epoch × Visit | 0.35 |
| Emotion × Epoch × Visit | 2.50 |
| Group × Emotion × Epoch × Visit | 0.55 |
|  |  |

****p* < .001.

##

## Neural Source Localization of Pupil Findings: fMRI Analysis

Since pupil dilation represents a summative index of cognitive and affective processes (Graur & Siegle, 2013), fMRI data were used from a subset of patients at baseline to localize the functional source of pupil dilation. While primary analyses were intentionally constrained to measures feasibly administered within a relatively inexpensive laboratory set-up, and for which both pre- and post-training data were available, fMRI data collected at baseline were used to provide potentially disambiguating data regarding the interpretation of primary pupil findings.

***Materials and Method.*** Of the 79 patients included in the main baseline analyses, 69 completed functional neuroimaging, and 62 had usable data from an identical SRET task completed subsequently (but also prior to treatment) in a 3Tesla Siemens Trio fMRI scanner. T2*-weighted images depicting BOLD contrast (TR=2000;TE=28;flip angle=73°; slices=38; FOV=200x200; 3.125x3.125x3.2mm voxels) were acquired and preprocessing steps were applied using Analysis of Functional Neuroimaging (AFNI), as previously described, using our laboratory’s standard methods (Price et al., 2018; Woody et al., 2019). Baseline-corrected contrast values during an early (2,000-6,000ms) and late (6,001-12,000ms) epoch of threat trials during fMRI were regressed on pupil dilation scores at early, intermediate, and late stages collected outside the scanner at a previous study visit. We used AFNI’s 3dRegAna command to identify clusters where brain response was significantly related to pupil response. Type 1 error for voxel-wise tests was controlled using contiguity thresholds derived based on the autocorrelation of the statistical maps (AFNI’s 3dClustsim with the recommended -acf method to provide accurate Type I error control; voxel-wise *p* < .005; map-wise *p* < .05).

**Results**. Pupil dilation during initial stages of threat trials was correlated with fMRI activation in the first half of the trial in a cluster located in the right middle frontal gyrus (MNI

space: x = 33, y = 3, z = 56; Figure S1). This region is situated more broadly within the lateral prefrontal cortex (lPFC) and is involved in neural circuits that are critical in cognitive control and modulating attention for emotional stimuli (Banich et al., 2009; Nee, Wager, & Jonides, 2007). These findings suggest that in the current sample, individual differences in pupil dilation in response to threat may better reflect top-down cognitive control processes and/or cognitive load rather than salience circuit driven arousal or affective responding. This is consistent with other work that has shown that activity in the middle frontal gyrus has been previously tied to pupil dilation during cognitive-affective tasks performed in the scanner and that it is a key player in a neural circuit that is modulated by ABM (Beevers, Clasen, Enock, & Schnyer, 2015).

*Figure S1.* Right middle frontal gyrus region tracking pupil dilation during threat trials.


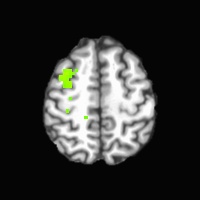

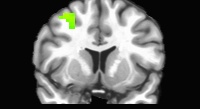

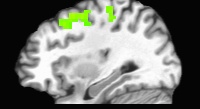


##

## Examination of Differences between Pupillary Response to Threat versus Neutral Words across the Time Course at Baseline

Mean stimulus-related pupil dilation was calculated across the 12,000ms trial, and pupillary responses were averaged across each emotion condition (neutral, threat). To assess whether there were significant differences between pupillary responses to neutral versus threat words across a continuous time course (rather than split into the *a priori* initial-intermediate-late time continuum), we examined pairwise differences at each sampled time point (collected at 60Hz). Differences were considered significant (*p* < .05) if they persisted for at least three consecutive time points. This time-region was identified using Guthrie and Buchwald’s technique to control for Type 1 error in timeseries data. Time points at which differences in pupillary response to neutral versus threat words are significant are highlighted in red and indicated by a bolded horizontal black line underneath the *X*-axis in Figure S1. The findings from these analyses are largely consistent with our examination of differences between pupillary responses to threat versus neutral words using the *a priori* defined initial-intermediate-late time continuum, providing additional support for this approach.


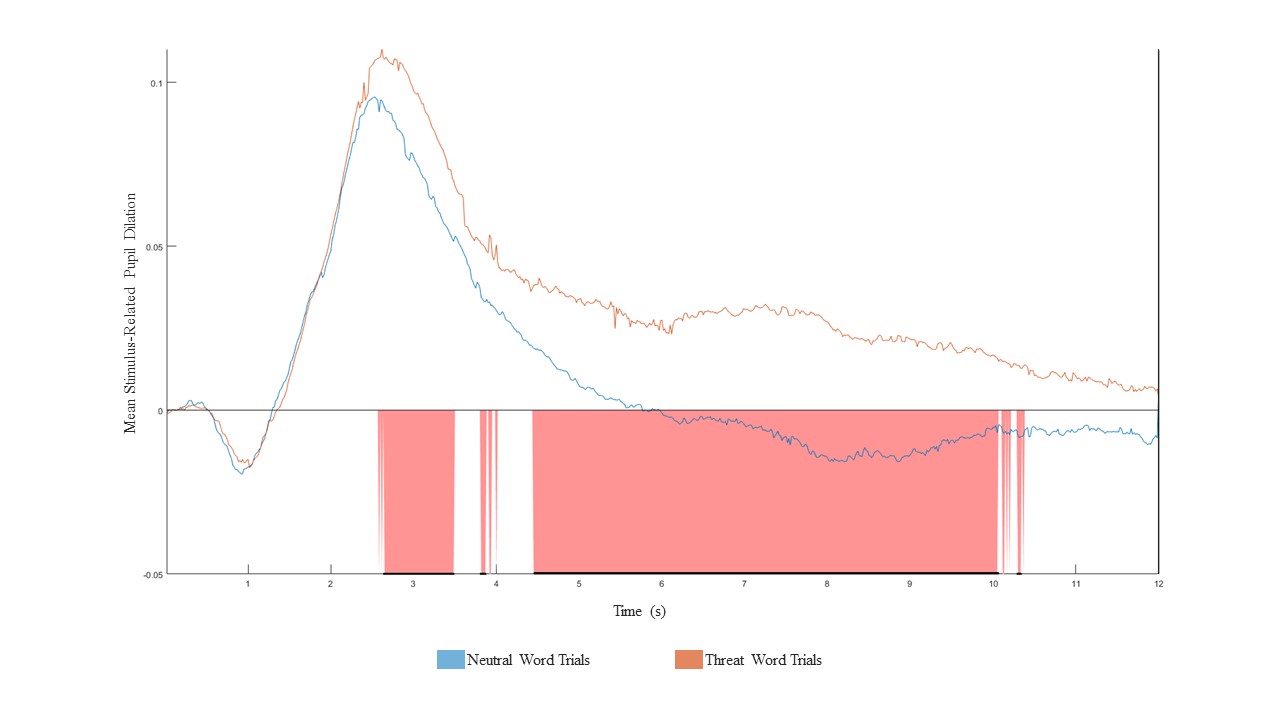
*Figure S2*. Differences between Pupillary Response to Threat versus Neutral Words across the Time Course.

Mean stimulus-related pupil dilation is plotted across the 12,000ms trial for both neutral and threat words. Significant pairwise differences are highlighted in red below the axis with bolded black lines showing time regions with enough consecutive tests (>3) to be considered significant (*p* < .05).

##

## Analysis of Secondary Clinical Outcome

In the main manuscript, findings demonstrated that patients who exhibited greater symptom improvement in self-reported vigilance in response to ABM also exhibited greater reductions in pupillary response to threat words during intermediate and late epochs of the trial. To determine if pupillary changes were specifically related to changes in self-reported vigilance (as indexed by the MASQ Anxious Arousal subscale), we conduct sensitivity analyses examining the MASQ General Distress: Anxiety subscale as a secondary clinical outcome. Among ABM patients, MASQ anxiety-specific general distress residual scores were not significantly related to reduced pupillary response to threat words during intermediate or late epochs of the trial (lowest *p* = .26).

**Supplemental References**

Banich, M. T., Mackiewicz, K. L., Depue, B. E., Whitmer, A. J., Miller, G. A., & Heller, W. (2009). Cognitive control mechanisms, emotion and memory: a neural perspective with implications for psychopathology. *Journal of Neuroscience Biobehavioral Reviews, 33*(5), 613-630.

Beevers, C. G., Clasen, P. C., Enock, P. M., & Schnyer, D. M. (2015). Attention bias modification for major depressive disorder: Effects on attention bias, resting state connectivity, and symptom change. *Journal of abnormal psychology, 124*(3), 463.

Blake, D. D., Weathers, F. W., Nagy, L. M., Kaloupek, D. G., Gusman, F. D., Charney, D. S., & Keane, T. M. (1995). The development of a clinician-administered PTSD scale. *Journal of traumatic stress, 8*(1), 75-90.

Clark, L. A., & Watson, D. (1991). Tripartite model of anxiety and depression: psychometric evidence and taxonomic implications. *Journal of abnormal psychology, 100*(3), 316.

Graur, S., & Siegle, G. (2013). Pupillary motility: bringing neuroscience to the psychiatry clinic of the future. *Current neurology and neuroscience reports, 13*(8), 365.

Jastak, S. R., & Wilkinson, G. S. (1984). *WRAT-R: Wide range achievement test-revised administration manual*: Jastak Associates.

Nee, D. E., Wager, T. D., & Jonides, J. (2007). Interference resolution: insights from a meta-analysis of neuroimaging tasks. *Cognitive, Affective, Behavioral Neuroscience, 7*(1), 1-17.

Price, R. B., Cummings, L., Gilchrist, D., Graur, S., Banihashemi, L., Kuo, S. S., & Siegle, G. J. (2018). Towards personalized, brain-based behavioral intervention for transdiagnostic anxiety: Transient neural responses to negative images predict outcomes following a targeted computer-based intervention. *Journal of consulting and clinical psychology, 86*(12), 1031-1045.

Spielberger, C. D. (2010). State‐Trait anxiety inventory. *The Corsini encyclopedia of psychology*, 1-1.

Üstün, T. B., Kostanjsek, N., Chatterji, S., & Rehm, J. (2010). *Measuring health and disability: Manual for WHO disability assessment schedule WHODAS 2.0*: World Health Organization.

Watson, D., Weber, K., Assenheimer, J. S., Clark, L. A., Strauss, M. E., & McCormick, R. A. (1995). Testing a tripartite model: I. Evaluating the convergent and discriminant validity of anxiety and depression symptom scales. *Journal of abnormal psychology, 104*(1), 3.

Woody, M. L., Yang, J. O., Cummings, L., Gilchrist, D., Graur, S., Siegle, G. J., & Price, R. B. (2019). Protracted amygdalar response predicts efficacy of a computer-based intervention targeting attentional patterns in transdiagnostic clinical anxiety. *Translational Psychiatry, 9*(1), 121.
